# Radiation dosimetry of [^68^Ga]PSMA-11 in low-risk prostate cancer patients

**DOI:** 10.1186/s40658-018-0239-2

**Published:** 2019-01-11

**Authors:** Kristina Sandgren, Lennart Johansson, Jan Axelsson, Joakim Jonsson, Mattias Ögren, Margareta Ögren, Martin Andersson, Sara Strandberg, Tufve Nyholm, Katrine Riklund, Anders Widmark

**Affiliations:** 1 Department of Radiation Sciences, Radiation Physics, Umeå University, 901 85 Umeå, Sweden; 2 Department of Radiation Sciences, Diagnostic Radiology, Umeå University, Umeå, Sweden; 3 Department of Medical Radiation Physics, ITM, Lund University, Malmö, Sweden

**Keywords:** Radiation dosimetry, [^68^Ga]PSMA-11, PSMA, PET-tracer, Prostate cancer, Absorbed dose and effective dose, Glu-NH-CO-NH-Lys(Ahx)-HBED-CC

## Abstract

**Background:**

^68^Ga-labeled Glu-NH-CO-NH-Lys(Ahx)-HBED-CC ([^68^Ga]PSMA-11) has been increasingly used to image prostate cancer using positron emission tomography (PET)/computed tomography (CT) both during diagnosis and treatment planning. It has been shown to be of clinical value for patients both in the primary and secondary stages of prostate cancer. The aim of this study was to determine the effective dose and organ doses from injection of [^68^Ga]PSMA-11 in a cohort of low-risk prostate cancer patients.

**Methods:**

Six low-risk prostate cancer patients were injected with 133–178 MBq [^68^Ga]PSMA-11 and examined with four PET/CT acquisitions from injection to 255 min post-injection. Urine was collected up to 4 h post-injection, and venous blood samples were drawn at 45 min, 85 min, 175 min, and 245 min post-injection. Kidneys, liver, lungs, spleen, salivary and lacrimal glands, and total body where delineated, and cumulated activities and absorbed organ doses calculated. The software IDAC-Dose 2.1 was used to calculate absorbed organ doses according to the International Commission on Radiological Protection (ICRP) publication 107 using specific absorbed fractions published in ICRP 133 and effective dose according to ICRP Publication 103. We also estimated the absorbed dose to the eye lenses using Monte Carlo methods.

**Results:**

[^68^Ga]PSMA-11 was rapidly cleared from the blood and accumulated preferentially in the kidneys and the liver. The substance has a biological half-life in blood of 6.5 min (91%) and 4.4 h (9%). The effective dose was calculated to 0.022 mSv/MBq. The kidneys received approximately 40 mGy after an injection with 160 MBq [^68^Ga]PSMA-11 while the lacrimal glands obtained an absorbed dose of 0.12 mGy per administered MBq. Regarding the eye lenses, the absorbed dose was low (0.0051 mGy/MBq).

**Conclusion:**

The effective dose for [^68^Ga]PSMA-11 is 0.022 mSv/MBq, where the kidneys and lacrimal glands receiving the highest organ dose.

## Background

Prostate cancer is one of the most commonly diagnosed malignant diseases. The increased prevalence is mainly attributed to the introduction of prostate-specific antigen (PSA) tests in the late 1980s, used for screening and diagnosis of non-palpable prostate cancers [1]. In addition to diagnosing more prostate cancer cases at an earlier age, a consequence of PSA-testing is an increased number of over-diagnosed and subsequently over-treated patients. Estimates show that more than one third of all PSA-detected prostate cancer cases would never have been discovered clinically nor harmed the patient during his lifetime [2].

Another biomarker for prostate cancer is prostate-specific membrane antigen (PSMA), which is a membrane-bound zinc protease encoded by the FOLH1 gene, shown to be a marker for a type of aggressive prostate cancer cells [3]. During the past couple of years, PSMA ligands have been increasingly used to image prostate cancer with positron emission tomography (PET)/computed tomography (CT). Several different PSMA ligands for PET imaging have been developed, either bound to the suitable PET radionuclides ^68^Ga (*t*_1/2_ = 68 min) or ^18^F (*t*_1/2_ = 110 min) [4]. The most commonly used ^68^Ga-labeled ligand is Glu-NH-CO-NH-Lys(Ahx)-HBED-CC ([^68^Ga]PSMA-11) and the theragnostic agents [^68^Ga]PSMA-617 and [^68^Ga]PSMA-I&T. ^18^F-labeled agents include [^18^F]DCFBC, [^18^F]DCFPyL, and [^18^F]PSMA 1007 [5]. At present, the most studied PSMA PET ligand is [^68^Ga]PSMA-11. [^68^Ga]PSMA-11 has consistently proved to be of considerable clinical value for patients both in the primary and secondary stages of prostate cancer [6–12].

Several research groups have investigated the radiation dosimetry of [^68^Ga]PSMA-11 [13–16]. However, despite the current surge in studies investigating the clinical impact of PSMA-PET for prostate cancer, to our knowledge, no prospective study has yet evaluated the radiation dosimetry of [^68^Ga]PSMA-11 in a cohort of low-risk prostate cancer patients. The aim of this paper was to determine the effective dose and organ doses after an injection of [^68^Ga]PSMA-11.

## Methods

The study was conducted as a sub-study within a non-commercial clinical trial (EudraCT no 2015-005046-55) on the diagnostic use of [^68^Ga]PSMA-11 in prostate cancer patients.

### Characteristics of participants

Six patients were included, all matching the following inclusion criteria: signed informed consent, older than 18 years, able to perform a PET/CT examination, and classified as a low-risk prostate cancer patient (TNM stage T1-T2; PSA < 10; Gleason score 6 or 7). The mean age of the patients was 68 years (range 62–72 years), the mean PSA level was 7.4 ng/mL (range 0.37–12.1 ng/mL), and all patients had biopsy-confirmed prostate cancers ranging between TNM stage T1a and T1c. The patient mean weight was 80 kg (range 67–90 kg).

All patients included in this study signed a written informed consent form allowing anonymized evaluation and publication of their data. This study was in accordance with the Helsinki Declaration and approved by the regional ethical review board at Umeå University, the local radiation safety committee at the University Hospital of Umeå, and the Swedish Medical Products Agency.

### [^68^Ga]PSMA-11

The production of [^68^Ga]PSMA-11 was done with an automatic synthesizer module with disposable cassettes (FASTlab developer, GE Healthcare) in a class C facility. Elution of ^68^GaCl_3_ from the ^68^Ge/^68^Ga generator (GalliaPharm, Eckert & Ziegler) was included as the first step of the production process, performed by the FASTlab. The whole eluate volume from the generator was passed through a solid phase cation extraction column, trapping the ^68^Ga^3+^. After washing, the trapped ^68^Ga^3+^ was eluted with 500 μL of a NaCl mixture and mixed with the precursor peptide HBED-CC-PSMA-11, dissolved in ammonium acetate buffer (pH 4.6). Binding of ^68^Ga with the HBED chelate in the precursor HBED-CC-PSMA-11 was performed by heating at 100 °C [17]. The product, [^68^Ga]PSMA-11, was purified using a reversed-phase solid phase extraction cartridge (Sep-Pak C-18 light, Waters), washed with water and eluted with 50% ethanol, buffered with sterile phosphate buffer (pH 7.4), and delivered through a sterile filter (0.2 μm) to the product vial. All productions passed quality control, where radiochemical purity, amount of peptide, and free ^68^Ga^3+^ were checked with high-performance liquid chromatography. Colloidal ^68^Ga was analyzed with thin-layer chromatography, and residual solvents were checked using gas chromatography.

### Imaging/scan protocol

All individuals received an intravenous bolus injection of 2 MBq/kg [^68^Ga]PSMA-11 (mean 155 MBq, range 133–178 MBq).

**Fig. 1:**
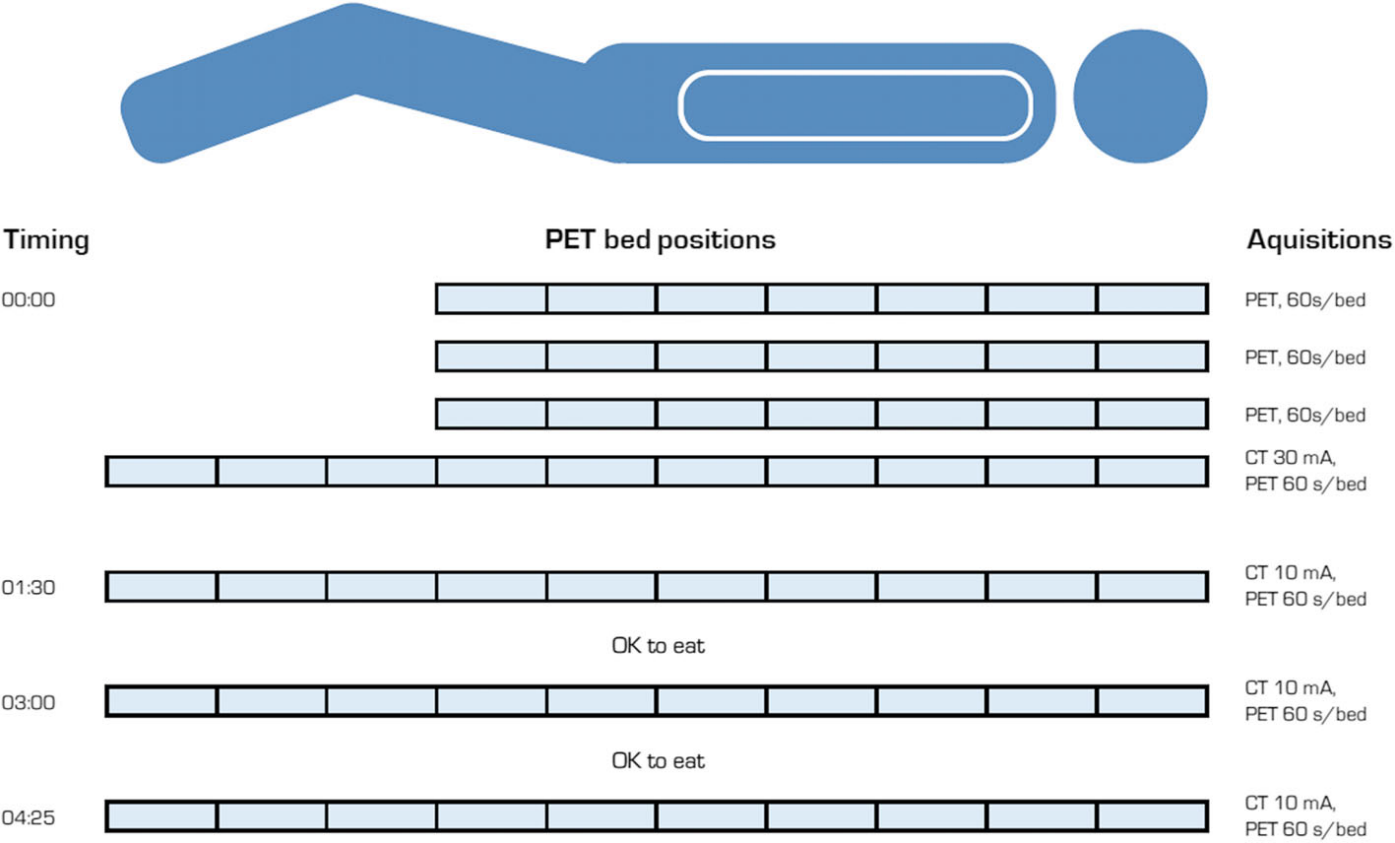
PET/CT scanning protocol. Illustration of the PET/CT scanning protocol. All PET scans were performed with 60 s/bed position. Immediately after injection, three head-to-thigh PET scans and one whole-body PET/CT scan were performed within the first 40 min. Additional whole-body PET/CT scans were performed at 1 h 30 min, 3 h, and 4 h 25 min after injection

The PET/CT scanning was performed at the University Hospital of Umeå on a Discovery 690 [18] (GE Healthcare, Milwaukee, WI, USA), in four sessions, summarized in Fig. 1. The first session, aimed to depict the fast tracer distribution, consisted of three head-to-thigh scans commencing at times 0, 10, and 20 min post-injection, followed by a head-to-toe whole-body scan approximately 30 min post-injection. The remaining three acquisitions were all head-to-toe scans, commencing at 90 min, 180 min, and 255 min post-injection. CT scans were acquired with 120 kV, 10 mA, 0.5 s/revolution, and 2.5-mm slice thickness, except for the whole-body scan at the first session which was acquired with 30 mA (Fig. 1) to provide adequate anatomical information. All PET scans were acquired using time-of-flight mode, with 60 s per bed position, and 11 slices bed overlap. PET images, corrected for attenuation and scatter, were reconstructed using the scanner implementation of the 3D iterative ordered subset expectation maximization (OSEM) reconstruction including time-of-flight (named VuePoint HD ViP) with 2 iterations, 24 subsets, 6.4-mm post-filter, and a 70-cm field-of-view, yielding a 128 × 128 matrix (5.5 × 5.5 × 3.27 mm^3^ voxels). PET images were decay-corrected to start of each scan.

### Blood and urine sampling

Urine was collected up to 4 h post-injection, and the total voided volume was weighed. From each voided volume, a 1-mL sample was weighed and radioactivity was measured using a well counter (NaI-scintillator), and the activity concentration in Bq/mL was calculated. The activity concentration and voided volume were used to calculate the total voided activity.

Venous blood samples were drawn at 45 min, 85 min, 175 min, and 245 min post-injection. The activity concentrations of these samples were measured in the abovementioned well counter. No metabolite analysis of the samples was performed, since this is beyond the scope of this dosimetry study.

### Absorbed dose calculations

Volumes of interest (VOIs) were delineated for the kidneys, liver, lungs, spleen, salivary glands, and total body by a medical physicist and a radiologist using RayStation (RaySearch Laboratories, Stockholm, Sweden). Using the MICE Toolkit (Nonpi Medical AB, Umea, Sweden), the median VOI activity-concentration (*C*_median_(*t*)) was extracted and used to calculate the fraction injected activity for each imaging session. For a specific VOI, the retention, *U*(*t*), in an organ with mass *M* [g], expressed as fraction of injected activity, is calculated as:

1 $$ U(t)=\frac{C_{\mathrm{median}}(t)\times M}{A_0} $$

where *A*_0_ is the administered activity [Bq] and *t* is the time after administration. The organ masses used are from the voxel phantom for adult males including blood content (International Commission on Radiological Protection (ICRP) Publication 133 [19]). The remaining activity was assumed to be homogeneously distributed in the rest of the body, and the remaining activity in the body could therefore be calculated by subtracting the activities in the specific organ, blood, urinary bladder content, and urine from the decay-corrected administered activity.

The uptake was fitted to a bi-exponential or mono-exponential function of time post-injection using the software SAAM II (The Epsilon Group, Charlottesville, VA, USA) [20]. This function is used to calculate the time-integrated activity coefficient (TIAC), i.e., total number of decays per administered activity. For the salivary glands, it was not possible to find an acceptable retention equation in this way—for those organs, the total number of decays was estimated directly from the uptake data as:

2 $$ \overset{\sim }{A}={\sum}_{i=1}^n\left(\frac{U(i)+U\left(i-1\right)}{2}\ \left(t(i)-t\left(i-1\right)\right)\right)+\frac{U(n)}{\lambda } $$

where *t*(0) and *U*(0) equal zero and *λ* is the physical decay constant (0.01 min^−1^) for the radionuclide. The cumulated activity in the urinary bladder was calculated in a similar way, using measured data for urine plus bladder content. ICRP uses 3.5-h voiding interval for calculating absorbed doses from radiopharmaceuticals [21]; thus, the activity in the bladder is zeroed at 3.5 and 7 h post-injection, before calculating the cumulated activity in accordance with Eq. 2.

The absorbed dose to the organs was calculated using the software IDAC-Dose 2.1 [22] which uses specific absorbed fractions for photons and electron from ICRP Publication 133 [19], and the calculation of effective dose was performed according to the ICRP Publication 103 [23].

**Fig. 2:**
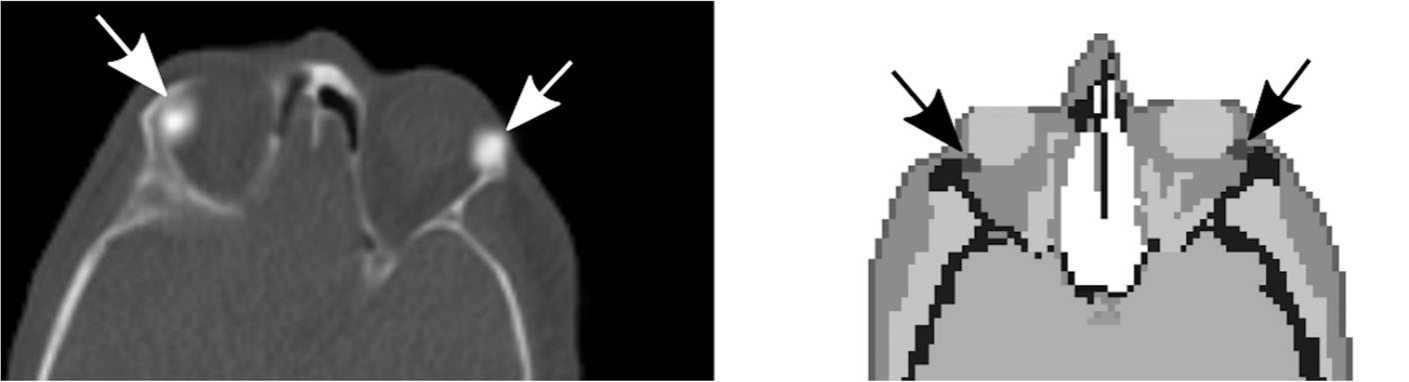
Lacrimal glands. PET/CT image of the [^68^Ga]PSMA-11 uptake in the lacrimal glands (left). One slice in the male adult voxel phantom with the lacrimal glands (at arrow points) on the lateral side of the orbits (right)

Because of the high uptake of [^68^Ga]PSMA-11 in the lacrimal glands, it was considered of interest to estimate the absorbed dose to the lacrimal glands and to evaluate their dose contribution to the eye lenses. To calculate the dose to the lacrimal glands, spherical shape was assumed, with a mass of 0.7 g each [24], using the “sphere module” in IDAC-Dose 2.1. They were added as a few voxels, located in the superior lateral region of each orbit in the phantom (Fig. 2). The lens of the eyes is a target organ in ICRP Publication 133 [19]; however, the lacrimal glands are not identified as source organs in the ICRP phantoms and an absorbed fraction is not presented in this report. Monte Carlo methods were therefore used to calculate absorbed fractions using the ICRP/ICRU voxel phantom for an adult male [25]. For the image activity concentration, the highest voxel was used, instead of the median. The reason for this is that the small size of the glands results in a significant “partial volume effect” [26]. To study the magnitude of this effect, a NEMA Image Quality phantom was scanned and it was found that for 10-mm spheres, similar in size to the glands, the maximum activity should be multiplied by a factor 2 to yield a good approximation of the actual activity concentration.

## Results

**Fig. 3:**
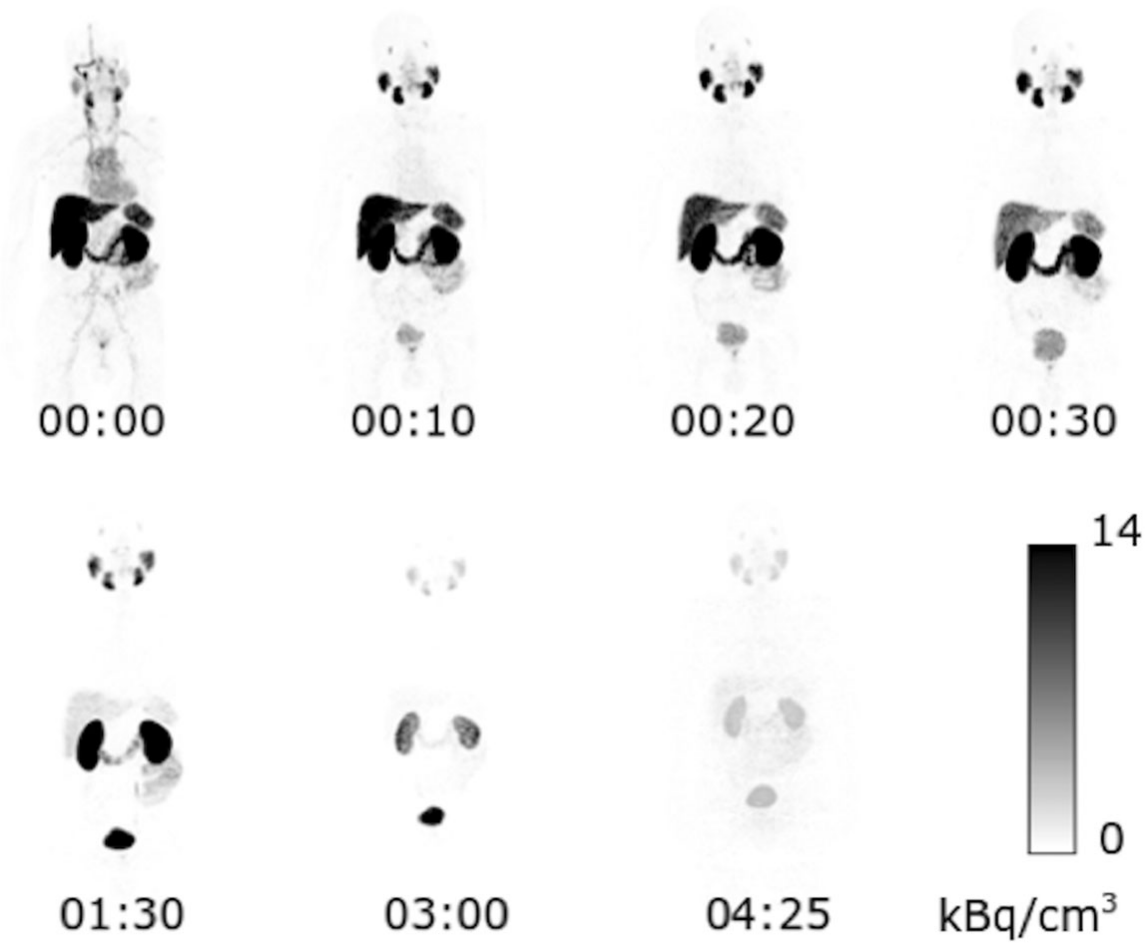
Whole-body maximum intensity projection. Whole-body maximum intensity projection from [^68^Ga]PSMA-11-PET scans of one participant at each of the seven scans

**Fig. 4:**
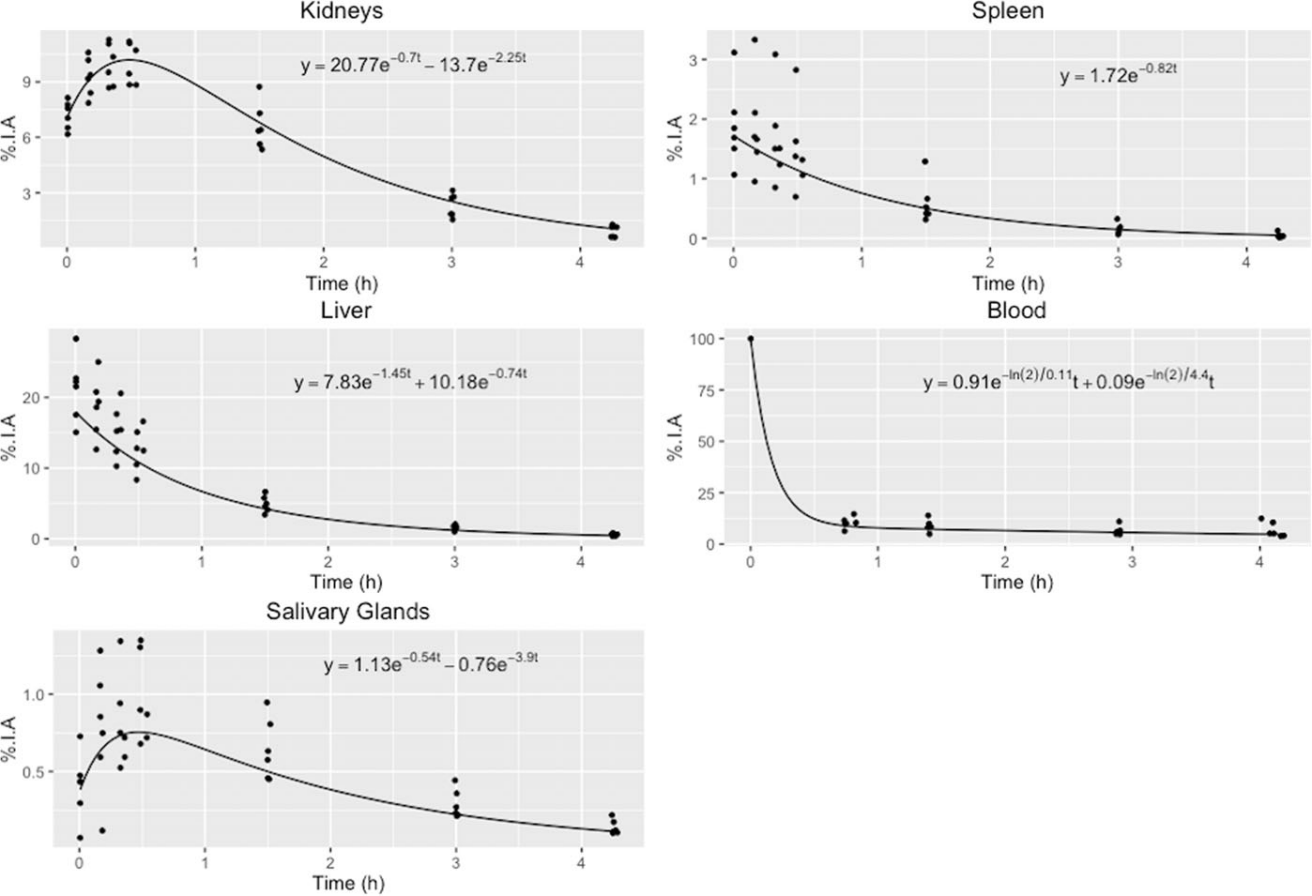
Injected activity. The median activity concentration of the six participants expressed in percent injected activity (%IA) for a selection of organs. The fitted bi-exponential or mono-exponential functions used can be seen in each graph

Intravenously injected [^68^Ga]PSMA-11 is rapidly cleared from the blood and is accumulated preferentially in the kidneys (7%), liver (15%), spleen (2%), and salivary glands (0.5%), see Fig. 3. The blood activity showed a bi-exponential behavior, containing a fast component with a 6.5-min half-life, and an additional slow 4.4-h half-life component reflecting biological clearance from blood; this is included as a source organ for the dose calculation. The median effective dose was calculated to be 0.022 mSv/MBq. TIACs for the organs used in the dose calculations can be seen in Table 1. Table 2 shows the median absorbed doses per administered activity unit. Organ activity uptake at different time points post-injection of [^68^Ga]PSMA-11 and their fitted curves are displayed in Fig. 4. The fitted blood curve was calculated on pooled normalized data and forced through the point 100% at time zero.

**Table 1:** Median and individual time-integrated activity coefficients of an injection with [^68^Ga]PSMA-11. The individual TIACs are calculated using the organ masses of the ICRP/ICRU voxel phantom

	Time-integrated activity coefficients						
Organ	Median [h]	P1	P2	P3	P4	P5	P6
Kidneys	0.22	0.22	0.27	0.19	0.23	0.19	0.26
Liver	0.23	0.26	0.15	0.22	0.23	0.19	0.31
Spleen	0.021	0.049	0.012	0.017	0.026	0.019	0.022
Salivary glands	0.020	0.036	0.020	0.021	0.017	0.030	0.017
Urinary bladder contents	0.11	0.12	0.094	0.13	0.041	0.16	0.050
Blood	0.27	0.32	0.26	0.36	0.22	0.25	0.28
Remainder	0.68	0.51	0.76	0.62	0.78	0.73	0.63
Lacrimal glands	0.00053	0.00075	0.00055	0.00038	0.00093	0.00052	0.00020

**Table 2:** The median values and the range of the absorbed doses per injected activity of [^68^Ga]PSMA-11 to organs for the six participants. Doses were calculated using the software IDAC-Dose 2.1

Organ	Absorbed dose coefficients	
	Median [mGy/MBq]	Range [mGy/MBq]
Kidneys	0.24	0.20–0.28
Liver	0.053	0.038–0.071
Lungs	0.016	0.013–0.017
Spleen	0.046	0.030–0.10
Salivary glands	0.089	0.074–0.15
Urinary bladder wall	0.057	0.028–0.084
Endosteum	0.011	0.0095–0.011
Red bone marrow	0.015	0.014–0.015
Lacrimal glands	0.11	0.043–0.2
Eye lenses	0.0051	0.0067–0.0054
Stomach wall	0.015	0.015–0.017
Colon wall	0.014	0.012–0.014
Esophagus	0.014	0.011–0.015
Skin	0.0067	0.0059–0.0069
Testes	0.0087	0.0074–0.0089
Thyroid	0.010	0.0090–0.010

The activity concentration in the voxels that were used to assess retention data included also activity in the blood. The obtained results in terms of cumulated activity thus also include activity in the blood that is present in the organ. The highest activity concentration is found in the kidneys, which therefore is the organ among those included in the calculation which obtains the highest absorbed dose. Typically, the kidneys will receive 0.24 mGy/MBq, resulting in 40 mGy after an injection of 160 MBq [^68^Ga]PSMA-11.

## Discussion

### Patient population and effective dose

According to ICRP Publication 103 [23], the effective dose should be calculated as an average of the equivalent dose calculated for men and women, preferably in healthy volunteers. However, due to ethical constraints, we choose to use a population of low-risk prostate cancer patients, and therefore, only the “male component” of the effective dose was calculated.

### Dose calculations

When the dose from diagnostic radiopharmaceuticals is estimated, the individual dose is of no or only minor interest. The effective dose is designed by ICRP to be calculated using the absorbed fractions derived from the standard phantom and should not be calculated using individual anatomical data. We have chosen to use the new ICRP/ICRU voxel phantom also when estimating the TIACs in the different organs. In this way, uncertainties in the determination of the individual volume of the organs are eliminated. It builds however on the assumption that the activity concentration in an organ is approximately constant independent of size. The method also requires that the remaining activity in the body is treated in a similar way. In our case, the remaining activity for each time-point has been calculated as decay corrected injected activity minus specified organs, blood, urinary bladder content, and urine. In that way, the activity in all parts of the voxel phantom will add up to the total body activity.

The new ICRP/ICRU voxel phantom was presented already in ICRP Publication 110 [25]; however, the specific absorbed fractions were not available for general use until ICRP Publication 133 [19] was published. There are some obvious differences from data that were normally used [25], which were based mainly on a mathematical phantom. In ICRP Publication 133 [19], absorbed fractions are given for electrons and beta-particles. This will influence the absorbed dose calculations for small organs and for organs with walls. In nuclear medicine, the absorbed dose to the urinary bladder wall is often high, due to activity in the bladder content. Applying this new electron-dose data to the bladder wall results in an absorbed dose that is typically half of the result from calculations with earlier used methods [25]. However, in the calculations, the bladder has a fixed volume, giving uncertainties in the approximation of bladders with varying volumes.

For comparison, absorbed doses have been calculated using a TIAC for the larger organs that were derived using a more conventional method, with measured volumes. The resulting dose for all those organs is well inside the range given in Table 2, except for the liver with a dose factor of 0.034 mGy/MBq. This discrepancy is explained by the fact that all our patients showed a liver mass approximately half of that of reference man, 800–1200 g compared to 2360 g, resulting in a TIAC of 0.10 h instead of 0.23 h.

### Lacrimal glands and eye lenses

The uptake shown in the lacrimal glands will result in a significant dose from ^68^Ga, approximately 1.19E−10 Gy/decay (428 mGy/MBq h), given that the organ is spherical with a mass of 0.7 g. Applying this approximation for the lacrimal glands means that this organ will obtain an absorbed dose of 0.12 mGy per administered megabecquerel. This is less than that for the kidneys, but the second highest organ dose. PSMA uptake in the lacrimal glands has been observed by others [16, 27–29]; however, there is little published data on absorbed dose to the lacrimal glands found in the literature, possibly partly due to the low cancer frequency in this organ [30] and the assumed low sensitivity to radiation. Furthermore, ICRP do not include the lacrimal glands in their different reports, not even in the Reference man report (ICRP Publication 89 [31]). A mass as low as 0.7 g for one gland means that the partial volume effect will affect the assessment of the total activity content considerably. To compensate for this, a NEMA phantom was measured on the same scanner to derive a correction factor, and it was found that the voxel showing the measured activity of the highest voxel should be multiplied by a factor of 2 to obtain the true activity concentration in the gland.

The lens of the eye is a radiation-sensitive organ in close connection to the lacrimal gland. The distance between the lacrimal gland and the lens of the eye is short, and the diameter of the eyeball is approximately 24 mm [32]. However, mean range for positrons produced from ^68^Ga decay (positron emission decay 89%, EC 11%) is 3.5 mm while the most energetic positrons can reach 9.2 mm [33]. Consequently, the dimensions of the eyeball are large enough to stop almost all except for the most energetic positrons before they reach the lens. The Monte Carlo calculations result in an absorbed dose factor for the lenses of the eyes with a source in the lacrimal glands is 5.37E−14 Gy/decay (0.192 mGy/MBq h in both glands). The major part of it (95%) is due to the 511 keV photons. This means that the contribution to the absorbed dose to the lens from the lacrimal glands is negligible in comparison with the dose from the rest of the body.

### Comparisons

Earlier studies [13–16] have also reached the conclusion that the kidneys are the organ obtaining the highest absorbed dose. The absorbed dose to the kidneys found in this study (0.24 mGy/MBq) is in line with the estimation by Afshar-Oromieh et al. [15] and Demirci et al. [16] who reported a kidney dose of 0.262 mGy/MBq and 0.246 mGy/MBq, respectively. It may, however, be noted that the TIACs reported by these authors are somewhat higher than ours, but a similar absorbed dose is obtained. The explanation is that they use a dose calculation program which is based on another phantom [34]. This phantom has a kidney mass of 299 g, while the mass of the kidneys in the ICRP/ICRU phantom is 422 g (incl. blood content).

Regarding the lacrimal glands, Demirci et al. [16] reported an absorbed dose of 0.04 ± 0.008 mGy/MBq which is in the lower region of our range (0.043–0.2 mGy/MBq). One possible cause to this deviation is that Demirci et al. [16] used the unit density sphere model, though did not provide the sphere volume, while our calculations assumed spherical mass of 0.7 g with a tissue equivalent material.

The effective dose was calculated using ICRP Publication 103 [23] and for comparison using ICRP Publication 60 [35]. Both methods resulted in an effective dose of 0.022 mSv/MBq, which is similar to the effective dose previously presented [13–16].

## Conclusions

The effective dose per injected activity from [^68^Ga]PSMA-11 was 0.022 mSv/MBq. Thus, an administered activity of 2 MBq/kg to a man weighting 80 kg gives an effective dose of 3.5 mSv. This can be compared to the effective dose from other PET-tracers used to image prostate cancer, e.g., [^18^F]Choline-PET (0.01 mSv/MBq) [36] and [^11^C]Acetate-PET (0.0049 mSv/MBq) [37], commonly administered using 3–4 MBq/kg ([^18^F]Choline) and 5 MBq/kg ([^11^C]Acetate). This reasonable effective dose indicates [^68^Ga]PSMA-11 to be a suitable substance for human imaging.
